# Antifungal Combination Therapy in Children with Cancer—A 4-Year Analysis of Real-Life Data of Two Major Pediatric Cancer Centers

**DOI:** 10.3390/jof7080604

**Published:** 2021-07-26

**Authors:** Stefan Schöning, Konrad Bochennek, Kathrin Gordon, Andreas H. Groll, Thomas Lehrnbecher

**Affiliations:** 1 Pediatric Hematology and Oncology, Hospital for Children and Adolescents, Johann Wolfgang Goethe-University, 60590 Frankfurt, Germany; stefan.schoening@kgu.de (S.S.); konrad.bochennek@kgu.de (K.B.); 2 Infectious Disease Research Program, Department of Pediatric Hematology and Oncology and Center for Bone Marrow Transplantation, University Children’s Hospital Münster, 48149 Münster, Germany; Kathrin.Gordon@ukmuenster.de (K.G.); andreas.groll@ukmuenster.de (A.H.G.)

**Keywords:** children, cancer, hematopoietic cell transplantation, invasive fungal disease, antifungal combination therapy

## Abstract

Clinical data on antifungal combination therapy are limited, in particular in the pediatric setting. We analyzed real-life data collected in two major pediatric cancer centers over a period of 4 years. Patients were identified in an observational study on children with acute leukemia and lymphoma or undergoing hematopoietic cell transplantation. Out of 438 patients, 19 patients received 21 episodes of antifungal combination therapy. Therapy was mostly started for sepsis (*n* = 5) or clinical deterioration with pulmonary infiltrates (*n* = 10), and less often for periorbital swelling with suspected mold infection (*n* = 2), clinical deterioration and new skin lesions, secondary antifungal prophylaxis, a persistently elevated galactomannan index, or as pre-emptive treatment (*n* = 1 each). Diagnostics revealed proven, probable, and possible invasive fungal disease in two, seven and four episodes, respectively. Most regimens included caspofungin (*n* = 19), and treatment was initiated as first line therapy in 10 episodes. The median duration was 13 days (4–46 days). Nine of the 13 patients with proven, probable, or possible invasive fungal disease survived, which was comparable to patients receiving antifungal monotherapy. Our analysis demonstrates that combination therapy has mainly been prescribed in selected immunocompromised patients with clinical deterioration due to suspected invasive fungal disease or those with sepsis, and is well tolerated. Future studies need to better characterize clinical settings in which patients may benefit from antifungal combination therapy.

## 1. Introduction

The outcome of children with leukemia and lymphoma as well as of children undergoing hematopoietic cell transplantation (HCT) has significantly improved over the last few decades [1,2]. However, despite the improvement of supportive care strategies, invasive fungal disease (IFD) still plays a major role in morbidity and mortality in these patient populations [3]. Although new and potent antifungal compounds including mold-active broad-spectrum triazoles and the new class of echinocandins have become available for the pediatric population, the mortality rate of IFD remains high. In that respect, a retrospective cohort study performed in the US identified 666 pediatric cases of invasive aspergillosis among 152,231 immunocompromised children and demonstrated that the fungal disease significantly prolonged the length of hospital stay and per-patient hospital charges but also significantly increased in-house mortality [4]. A recent analysis of children with acute lymphoblastic leukemia (ALL) revealed that the rate of infection-related death of 2.4% was mainly due to IFD [5]. In this study, proven IFDs were caused mostly by *Candida* spp. (*n* = 20) and less often by *Aspergillus fumigatus* (*n* = 6) and mucormycetes (*n* = 2), whereas a study of children with acute myeloblastic leukemia (AML) reported on a similar incidence of *Candida* and *Aspergilus* infections [6].

As in other settings of antimicrobial therapy, combination therapy is conceptually attractive, and the strategy of antifungal combination therapy is supported by in vitro data and animal studies [7,8]. Unfortunately, in the clinical setting, data on the use of antifungal combination therapy are scarce and conflicting, in particular in children and adolescents [9,10,11,12]. As randomized studies on antifungal combination therapy are difficult to perform and data are complex to interpret due to multiple confounding factors, we thought to analyze real-life data collected in two major pediatric cancer centers in Germany over a period of 4 years.

## 2. Patients and Methods

Children and adolescents up to the age of 18 years receiving combination antifungal therapy were identified from a prospective observational study (DRKS00006341), which included all patients diagnosed between 1 April 2014 and 31 March 2018 with de novo ALL, AML, relapse of acute leukemia, non-Hodgkin lymphoma (NHL), or undergoing allogeneic HCT. All patients were treated according to Berlin–Frankfurt–Münster (BFM)-based protocols at the University Children Hospitals of Frankfurt or Münster, Germany. Both centers routinely perform diagnostics such as imaging studies or culture and non-culture based microbiologic tests such as the assessment of galactomannan (Platelia™ Aspergillus Ag, Bio-Rad, Munich, Germany) in children at highest risk for IFD, e.g., in persistently febrile neutropenic patients not responding to broad-spectrum antibiotics after 96 h or in patients with any clinical sign or symptom consistent with IFD [13,14]. These patients also received empirical or pre-emptive mold-active antifungal therapy, respectively, whereas antifungal prophylaxis was instituted according to local standard operating procedures.

Data collection was performed using an electronic database (secuTrial^®,^ Berlin, Germany) and included demographic data, disease characteristics, data on laboratory diagnostic studies and imaging as well as information on antifungal drug use. Neutropenia was defined as an absolute neutrophil count ≤ 500/mm^3^ [15]. Invasive fungal disease was defined as proven, probable, and possible infection according to the revised definitions by the EORTC/MSG consensus group [16]. Briefly, proven IFD was defined by the detection of a fungus by culture in blood or an otherwise sterile compartment, or histopathological evidence of fungal elements in affected tissue. Probable IFD required the presence of host factors (e.g., severe and prolonged neutropenia, allogeneic HCT), clinical criteria (e.g., lower respiratory tract infection with computerized tomography (CT) imaging demonstrating lesions suggestive of an IFD), and mycological criteria (e.g., culture of a mold in sputum or broncho-alveolar lavage (BAL), detection of galactomannan (GM) in serum (optical density index of >1.0 [one sample] or >0.5 [two samples]) or BAL (cut-off 1.0)). Positive testing by PCR was not included as a criterion of probable IFD. Patients with appropriate host factors and with sufficient clinical evidence consistent for IFD, but without mycological support, were categorized as patients with possible IFD [16].

Last follow-up was at 12 months after the end of intensive chemotherapy or the date of allogeneic HCT and included the current status on relapse/disease-free survival, death and, in case of the occurrence of an IFD, the outcome of the infection. Detailed analysis of the response to combination therapy (e.g., complete or partial response) was not included as an endpoint, as most patients received multimodal antifungal treatment (e.g., granulocyte transfusions).

Statistics were descriptive with the exception of the comparison of laboratory values, where paired Student’s *t*-test was used. A *p* value of ≤0.5 (two-sided) was considered statistically significant.

The study was reviewed and approved by the local ethical committees of Frankfurt (348/13) and Münster (2014-048-b-S).

## 3. Results

Out of a total of 438 patients, 19 (4.3%) (14 boys, 5 girls, median age 11 years (range 0.4–17)) received 21 episodes of antifungal combination therapy (monotherapy: 239 episodes in 165 patients). Combination therapy was administered in 16 episodes during HCT, and in five episodes during intensive chemotherapy (Table 1).

The indication for instituting antifungal combination therapy was as follows: sepsis in five episodes, clinical deterioration with pulmonary infiltrates in 10 episodes, periorbital swelling with suspected mold infection of the paranasal sinuses in two episodes, clinical deterioration and new skin lesions in one episode, secondary antifungal prophylaxis, a persistently elevated galactomannan index, and pre-emptive treatment in one episode each (Table 2).

Diagnostic tests revealed proven invasive aspergillosis (biopsy: *A. fumigatus* (macroscopic and microscopic characteristics), sensitive to amphotericin B, caspofungin, and voriconazole (antibiotic gradient tests: Etest^®^, bioMérieux, Nürtingen, Germany)) and *C. krusei* blood stream infection (species identification by matrix-assisted laser desorption/ionization time-of-flight analysis (MALDI-TOF); sensitive to amphotericin B, caspofungin, micafungin and voriconazole (VITEK-2 [bioMérieux, Nürtingen, Germany])) in one patient each, probable invasive aspergillosis in seven patients, and possible invasive mold infection/invasive aspergillosis in two patients each. In eight episodes, there was no evidence of a fungal infection (Table 2).

Antifungal combination therapy consisted of 11 episodes of caspofungin and liposomal amphotericin B (LAMB), five episodes of caspofungin and voriconazole, and four episodes of voriconazole and LAMB (Table 2). In one episode each, caspofungin and posaconazole, LAMB and posaconazole, micafungin and voriconazole, caspofungin and micafungin or a combination of caspofungin, voriconazole, LAMB and posaconazole was given. In three patients, the therapeutic regimen of antifungal combination therapy was modified within a specific episode. The median duration (range) of antifungal combination therapy was 13 days (4–46 days) (Table 2).

Antifungal combination therapy was started as first line therapy in 10 episodes, and given after prior antifungal monotherapy in 11 episodes. Monotherapy prior to antifungal combination therapy was administered over a median of 15 days (range 2–125 days), and consisted of LAMB (*n* = 6), micafungin (*n* = 4), voriconazole (*n* = 2), or caspofungin (*n* = 1). Two patients received sequential monotherapy with micafungin and LAMB and voriconazole and LAMB, respectively. 

After the cessation of antifungal combination therapy, no further antifungal therapy was given in six episodes. In 15 episodes, antifungal monotherapy was administered after the cessation of antifungal combination therapy, and consisted of voriconazole (*n* = 5), caspofungin (*n* = 4), posaconazole (*n* = 3), LAMB (*n* = 2) or micafungin (*n* = 1), which were given for a median of 38 days (range of 4–176 days). The patient with proven aspergillosis died while receiving antifungal combination therapy, and the patient with blood stream infection to *C. krusei* received caspofungin monotherapy after the cessation of antifungal combination therapy. Monotherapy after antifungal combination therapy was given in five out of seven patients with probable IFD (voriconazole in three episodes, micafungin and posaconazole in one episode each), and in three out of four patients with possible IFD (two patients receiving LAMB, one patient receiving voriconazole).

Fourteen out of the 19 patients survived. One patient died due to invasive aspergillosis, three others due to multifactorial infectious problems including IFD, and one due to progression of the underlying malignancy. When only analyzing patients with proven and probable IFD, five of the nine patients survived; of the 13 patients with proven, probable, or possible IFD, nine survived (Table 2). These outcomes were comparable with those of patients receiving antifungal monotherapy for proven, probable, or possible IFD (Table 3).

**Figure 1:**
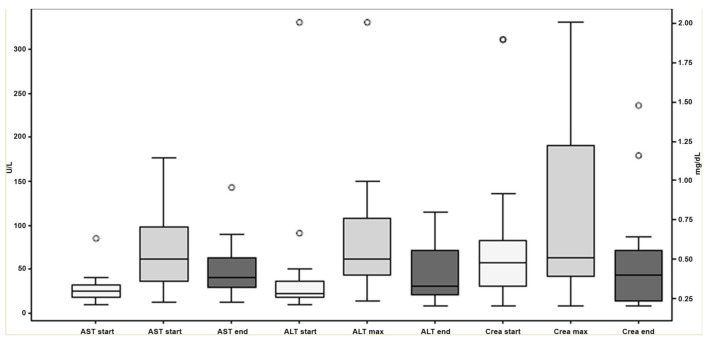
Plasma levels of aspartate transaminase (AST), alanine transaminase (ALT) and creatinine at baseline (white columns, start), maximum values during antifungal combination therapy (light grey columns, max), and at end of antifungal combination therapy (dark grey columns, end). The bars indicate the median, the boxes 25th and 75th percentiles and the whiskers the range. Outliers are indicated by the circles.

In none of the patients, antifungal combination therapy was prematurely discontinued due to adverse events. The median serum creatinine value at baseline was 0.48 mg/dL (range, 0.2–1.9 mg/dL), and was significantly lower than the maximum level during combination therapy (median (range) 0.51 mg/dL (0.2–2.0 mg/dL); *p* < 0.05) (Figure 1). Similarly, the maximum serum levels of the hepatic transaminases aspartate transaminase (AST) and alanine transaminase (ALT) during combination therapy (median (range) 61 U/L (12–177 U/L) and 62 U/L (14–331 U/L), respectively) were significantly higher than corresponding baseline levels (median (range) 25 U/L (9–85 U/L) and 22 U/L (9–331 U/L), respectively; *p* < 0.01 each). Whereas the levels of creatinine and ALT assessed at the end of treatment did not significantly differ from baseline levels, the levels of AST assessed at the end of treatment (median (range) 41 UL (12–143 UL)) were significantly higher than baseline levels (*p* < 0.05).

## 4. Discussion

Antifungal combination therapy may be a valid treatment option for a subpopulation of patients with hematological malignancies as it offers a number of potential advantages. For example, various in vitro and in vivo studies have demonstrated that combinations of antifungal agents can broaden the antifungal coverage, fasten the onset of antifungal efficacy, increase the fungicidal effect and decrease the risk of the development of resistance [17]. In a murine model of experimental pulmonary aspergillosis, the combination of the echinocandin micafungin and the triazole ravuconazole significantly decreased mortality and fungal burden [7], and the broad-spectrum triazole posaconazole exhibits in vitro and in vivo synergy with caspofungin against *C. albicans*, also affecting echinocandin-resistant isolates [8]. In the clinical setting, however, the impact of combination therapy on outcome is not clear. Whereas a systematic review and meta-analysis demonstrated that antifungal combination therapy may improve outcome in acute invasive aspergillosis [18], a large randomized, comparative clinical trial investigating the combination of voriconazole plus anidulafungin versus voriconazole as the standard of care for primary treatment of invasive aspergillosis in adult patients failed to demonstrate the superiority of the combination therapy [12]. Nevertheless, an unscheduled subgroup analysis of patients with probable invasive aspergillosis in whom the microbiological diagnosis was made on the basis of galactomannan results showed improved survival in the combination cohort, lending support for clinical benefit.

According to a survey conducted among pediatric units in the UK, first line combination antifungal therapy is rarely prescribed [19]. The most common reasons for antifungal combination therapy were disseminated invasive aspergillosis (77%) and coverage of a period of possible insufficient levels of azole antifungals (46%), whereas initial empiric treatment when diagnostic test results were awaited (15%) or the treatment of critically ill patients (8%) were less frequently named. This is in contrast to our analysis of real-life data, which demonstrated that clinical deterioration (48%) and empirical treatment for sepsis (24%) were the most frequent reasons to start antifungal combination therapy. However, it has to be noted that typical signs of pulmonary invasive fungal disease are often missing in immunocompromised children, and a recent meta-analysis demonstrated wide ranges for the specificity, sensitivity, and positive predictive value of galactomannan in the pediatric setting [13,20]. Clinical suspicion of non-*Aspergillus* molds such as periorbital swelling and skin lesions, both of which may indicate mucormycosis or the presence of a rare mold, led in 15% of the episodes to the institution of antifungal combination therapy. Current pediatric specific guidelines by the ECIL group assigned a CIIt and a CIII recommendation for the institution of combination therapy in invasive aspergillosis and mucormycosis, respectively (C: marginally supported, IIt case–control or case series, transferred evidence from adults, III expert opinion) [13]. In all five patients in whom antifungal combination therapy was instituted as they were septic, therapy was de-escalated after a median of 6 days (range, 4–14). Antifungal combination therapy was started first-line in 10 of 21 episodes, whereas in 11 episodes, prior monotherapy was either intensified by adding another antifungal agent (six episodes) or was replaced by the combination of two different compounds (five episodes). This corroborates the data of a multicenter surveillance study, where 46% of the 31 pediatric and 54 adult patients received a sequential antifungal therapy with the addition of a new drug to the previous monotherapy, and 54% of the patients were treated with two new antifungal agents [21]. Similar to our findings, caspofungin-based antifungal combination therapy was given in two thirds of the patients, and the median duration of antifungal combination therapy was 19 days, which is comparable to our results (13 days) [21]. 

In a retrospective study in 40 children and adolescents with cancer and probable or documented invasive aspergillosis, caspofungin-based combination therapy resulted in a favorable response in 53% of the patients and a 100-day survival rate of 70% [10]. This is comparable to our data, where out of the nine patients with proven/probable IFD, four died and five survived. However, it is important to note that in our analysis, patients receiving antifungal monotherapy for proven or probable IFD had a similar outcome with 8 out of 12 patients surviving (Table 3). Our data are supported by a prospective cohort study in 131 immunocompromised children with IFD, mostly invasive aspergillosis, and a retrospective study involving 159 adult patients with hematological malignancies and invasive aspergillosis, which demonstrated that antifungal combination therapy did not result in improved outcome [9,22]. We acknowledge the potential bias of our analysis because, as it is the fact in all non-randomized studies, patients who seem to be very ill will receive combination therapy, whereas the others who are judged as not that ill are treated with monotherapy. Importantly, the pivotal randomized clinical trial comparing voriconazole versus the combination of voriconazole plus anidulafungin for primary treatment of invasive aspergillosis did not show the superiority of the combination therapy [12]. However, although the study was not powered to detect meaningful differences in subsets of patients, higher survival rates were observed in specific subgroups of patients, which might suggest that some patients could benefit from combination antifungal therapy.

Conflicting results were reported regarding the safety of antifungal combination therapy. Whereas in one study antifungal combination therapy was associated with an increased risk of adverse events (risk ratio, 1.98; *p* = 0.031), others reported that 22% of the patients experienced only mild and reversible adverse events such as hypokalemia, the elevation of liver enzymes or a creatinine increase, which corroborates our results [9,21]. However, whether or not the changes in creatinine and liver enzyme values have to be attributed to combination antifungal therapy in these severely ill patients remains unclear. 

In conclusion, our analysis demonstrates that combination therapy has mainly been prescribed in a small number of selected immunocompromised patients with clinical deterioration due to suspected invasive fungal infection or those with sepsis, and that antifungal combination therapy is well tolerated. Prospective trials are difficult to perform as they are costly and are difficult to interpret due to the multitude of confounding factors, but our data may contribute to meta-analyses in order to shed light on the issues of antifungal combination therapy.

## Figures and Tables

**Table 1:** Demographics of 19 pediatric patients receiving antifungal combination therapy.

Patient #	Age (y)/Sex	Underlying Malignancy	Therapy	Neutrophil Count Prior to Combination Therapy (mm^3^)
1	12/boy	ALL	HCT	0
2	4/boy	Immunodeficiency	HCT	500
3	11/boy	Rhabdomyosarcoma	HCT	130
4	17/girl	ALL	Chemotherapy	70
5	6/boy	AML	Chemotherapy and HCT	110
6	14/boy	AML	HCT	20
7	17/boy	ALL	HCT	100
8	14/girl	DBA	HCT	200
9	9/boy	ALL	HCT/HCT	3100
10	8/boy	Fanconi anemia	HCT	2340
11	5/boy	ALL	Chemotherapy	20
12	9/girl	AML	HCT	20
13	1/boy	AML	Chemotherapy	10
14	0.4/girl	Immunodeficiency	HCT	110
15	14/boy	MDS	HCT	1260
16	15/boy	Aplastic anemia	HCT	18,000
17	17/boy	ALL	Chemotherapy	50
18	13/boy	Aplastic anemia	HCT	0
19	5/girl	ALL	HCT	2110

# patient number; y, year; ALL, acute lymphoblastic leukemia; AML, acute myeloid leukemia; DBA, diamond blackfan anemia; MDS, myelodysplastic syndrome; HCT, hematopoietic cell transplantation.

**Table 2:** Characteristics of antifungal combination therapy, clinical findings and outcome.

	Antifungal Treatment Prior to Combination Therapy (Days)	Indication for Combination Therapy	IFD (Fungal Pathogen)	Combination Therapy		Antifungal Treatment after Combination Therapy (Days)	Outcome
Patient #				Agents	Duration (Days)		
1	None	sepsis	no	caspo + LAMB	14	monotherapy caspo (5)	alive
2	monotherapy LAMB (44), vori (81)	clinical deterioration, pulmonary infiltrates	proven invasive aspergillosis	vori + LAMB	10	none	death (invasive aspergillosis)
3	monotherapy mica (14), LAMB (14)	clinical deterioration, pulmonary infiltrates	proven (C.krusei)	caspo + vori	14	monotherapy caspo (15)	death (multifactorial)
4	None	periorbital swelling	no	caspo + LAMB	4	none	alive
5	monotherapy mica (22)	clinical deterioration, pulmonary infiltrates	probable invasive aspergillosis	caspo + mica	12	monotherapy vori (84)	alive
				caspo + vori	21		
5 (second episode)	monotherapy LAMB (4)	clinical deterioration, pulmonary infiltrates	probable invasive aspergillosis	caspo + LAMB	30	monotherapy mica (28)	alive
6	monotherapy caspo (2)	sepsis	no	caspo + LAMB	6	monotherapy caspo (4)	alive
7	monotherapy LAMB (14)	sepsis	no	caspo + LAMB	4	none	alive
8	monotherapy mica (31)	persistent positive galactomannan index	possible (aspergilosis)	caspo + vori	26	monotherapy vori (176)	alive
				mica + vori	11		
				caspo + vori	18		
9	None	secondary prophylaxis	probable invasive aspergillosis	caspo + LAMB	19	monotherapy vori (60)	alive
9 (second episode)	None	clinical deterioration/progress pulmonary infiltrates	probable invasive aspergillosis	casp + LAMB	16		death (multifactorial)
10	monotherapy vori (38)	periorbital swelling	no	caspo + posa	7	monotherapy posa (42)	alive
11	None	sepsis, skin infiltrates	no	LAMB + posa	17	monotherapy vori (19)	alive
12	None	clinical deterioration, pulmonary infiltrates	possible (mold)	caspo + LAMB	10	monotherapy LAMB (48)	alive
13	None	pre-emptive therapy, febrile neutropenia	possible (aspergillosis)	vori + LAMB	46	monotherapy LAMB (52)	alive
14	None	sepsis	no	caspo + LAMB	13	monotherapy caspo (30)	death (unrelated to infection)
15	monotherapy mica (15)	clinical deterioration, pulmonary infiltrates	possible (mold)	caspo + vori	10	none	alive
16	monotherapy LAMB (12)	sepsis	no	caspo + LAMB	4	monotherapy caspo (33)	alive
17	None	clinical deterioration, infiltrates lung and cns	probable invasive aspergillosis	vori + LAMB	9	monotherapy vori (11)	alive
18	None	clinical deterioration, pulmonary infiltrates	probable invasive aspergillosis	caspo + LAMB	6	monotherapy posa (144)	alive
				vori + LAMB	5		
19	monotherapy LAMB (4)	clinical deterioration, pulmonary infiltrates	probable invasive aspergillosis	caspo + vori + LAMB + posa	24	none	death (multifactorial)

# patient number; LAMB, liposomal amphotericin B; mica, micafungin; caspo, caspofungin; vori, voriconazole; posa, posaconazole.

**Table 3:** Outcome of patients receiving antifungal monotherapy or antifungal combination therapy.

		Antifungal Monotherapy	Antifungal Combination Therapy
Proven/probable IFD		12	9
	Chemotherapy		
	alive	5	2
	dead	1	0
	HCT		
	alive	3	3
	dead	3	4
Possible IFD		21	4
	Chemotherapy		
	alive	12	1
	dead	1	0
	HCT		
	alive	6	3
	dead	2	0
No IFD		132	8
	Chemotherapy		
	alive	38	0
	dead	2	1
	HCT		
	alive	81	7
	dead	11	0

IFD, invasive fungal disease; HCT, hematopoietic cell transplantation.

## Data Availability

Not applicable.
